# Relationship Between Pulse Oximetry Perfusion Index and Anesthetic Brachial Plexus Block for Upper Limb Surgery in Adults

**DOI:** 10.7759/cureus.99394

**Published:** 2025-12-16

**Authors:** JD Bolívar, Rodolfo Carlos Sabogal, Valentina Palacio-Arango, Sebastian Robledo

**Affiliations:** 1 Anesthesiology and Critical Care, Clínica Especializada EMMSA, Medellín, COL; 2 Anesthesiology and Critical Care, Clínica Medihelp Services, Cartagena, COL; 3 General Medicine, Clínica Especializada EMMSA, Medellín, COL

**Keywords:** anesthesia, brachial plexus, perfusion index, pulse oximetry, upper limb

## Abstract

Introduction

Brachial plexus block is widely used as a regional anesthetic technique in upper extremity surgery. However, its routine assessment using clinical criteria, such as sensation and motor function, is limited by the subjective nature of the assessment and the dependence on the patient. The perfusion index, obtained by pulse oximetry, could be an objective, early method of predicting block success.

Objective

To establish the relationship between the increase in perfusion index and the success of anesthetic brachial plexus block in adult patients undergoing upper extremity surgery.

Methods

An observational, descriptive, cross-sectional study was conducted with 82 adult patients undergoing upper extremity surgery with anesthetic brachial plexus block using four different approaches. Block success was assessed using the Modified Bromage Scale at five, 10, and 15 minutes. The perfusion index was measured with pulse oximetry before the block and at the same intervals afterward. Odds ratios with Haldane-Anscombe correction and the Mantel-Haenszel test were used to adjust for confounders.

Results

A progressive increase in the perfusion index was observed at five, 10, and 15 minutes after the block. A value less than 3 had a negative predictive value of 100% for predicting anesthetic failure. At 10 minutes, a mean perfusion index of 4.96, a 6.2-fold increase over baseline, was associated with successful blockade, reaching a success rate of 73.2% at 15 minutes.

Conclusions

The perfusion index proved to be an objective and sensitive tool for predicting the success of brachial plexus blockade, allowing for earlier and more accurate assessment than traditional clinical signs. Its implementation could optimize anesthetic decision-making and reduce response times to ineffective blocks.

## Introduction

Due to its anatomical and functional disposition, the upper limb is susceptible to trauma from various mechanisms, which can result in disability or even life-threatening injuries. According to global statistics, between 10% and 40% of emergency department visits are related to upper limb trauma. In Colombia, accurate data on upper limb surgeries is underreported; however, local data indicate that 25,646 patients were treated for upper limb trauma in Medellín emergency departments between January and December 2016 [1]. These injuries may require surgical intervention, which can be performed under general or regional anesthesia. In this context, brachial plexus block has become one of the main regional anesthesia techniques, especially for procedures such as fracture reduction and internal fixation [2].

Regional anesthesia by brachial plexus block is superior to general anesthesia in terms of postoperative analgesia and reduction of opioid-related adverse effects [3], such as respiratory depression mediated by μ and δ receptors through direct inhibition of rhythm-generating respiratory neurons in the pre-Boetzinger complex [4].

During the neurostimulation era, different approaches for brachial plexus block were described depending on the cutaneous entry point and needle direction, such as the Winnie technique, with a high incidence of complications and side effects. However, ultrasound-guided techniques have renewed interest in periclavicular approaches through supra- or infraclavicular access because they reduce the risk of pleural puncture, pneumothorax, symptomatic hemidiaphragmatic paresis, Horner's syndrome, vascular puncture, and transient sensory deficit [5,6].

Local anesthetics are used for brachial plexus blocks. The choice of anesthetic depends on the onset and duration of its effects, the degree of motor blockade it produces, and its toxicity profile. These factors are influenced by the dose, concentration, and volume injected. For example, the use of bupivacaine has an intermediate onset of action greater than 10 minutes with a duration of effect greater than 60 minutes [7]. In addition, local anesthetics modulate the inflammatory response by inhibiting proinflammatory cascades, such as the mitogen-activated protein kinase (MAPK) pathway, as well as G protein-coupled receptors. They also reduce sensitization of the nervous system to tissue injury, thereby reducing hyperalgesia [8]. When administered around nerves, these agents temporarily block the function of the sympathetic nervous system in vascular smooth muscle, producing vasodilation depending on the anesthetic's concentration and type [9].

The success of peripheral nerve blockade is usually evaluated using sensory and motor function tests. However, these methods are subjective and time-consuming, and they depend on patient cooperation [10]. Therefore, research has been conducted to establish objective methods for assessing block quality, such as the use of the perfusion index obtained by pulse oximetry. This index represents the numerical ratio between pulsatile and non-pulsatile blood flow, as measured by a pulse oximeter. Currently, there is no defined cutoff value that accurately predicts a successful nerve block [11].

An observational study of 63 adult patients, aged 18-73 years and scheduled for elective upper- or lower-limb surgery under regional anesthesia, concluded that the pulse oximetry perfusion index is a simple, objective, noninvasive technique with high specificity and sensitivity for assessing the success or failure of regional blocks compared with conventional, sensitivity-based assessments (P ≤ 0.05, area ≥ 0.9, sensitivity ≥ 90%, and specificity ≥ 90%) [12].

Kus et al. conducted a study in 46 patients undergoing elective hand, wrist, and forearm surgery. They found that the perfusion index measured with pulse oximetry predicts the success of brachial plexus block using the infraclavicular approach 10 minutes after the anesthetic is administered [13]. Another study evaluated the relationship between the perfusion index and the success of brachial plexus block in 65 patients undergoing arm surgery with an ultrasound-guided supraclavicular approach. The study reported that a 3.25-fold increase in the perfusion index, compared to the baseline value 10 minutes after the procedure, correlated with 96.67% sensitivity and 100% specificity as an indicator of a successful block [10].

Finally, De Buono et al. conducted an observational study of 24 patients scheduled for extremity surgery. They concluded that the perfusion index reliably indicates successful nerve block when its baseline value triples five minutes after brachial plexus anesthesia using the interscalene approach [14].

Given that brachial plexus block produces physiological changes such as vasodilation and increased regional blood flow, which precede the loss of sensation and mobility in the limb, and that these changes can be monitored through the perfusion index, this study was proposed. The main objective is to establish the relationship between the increase in the perfusion index measured by pulse oximetry and the success of anesthetic brachial plexus block in adults undergoing upper extremity surgery.

## Materials and methods

A descriptive, observational, cross-sectional study was conducted at a single center: the Hospital Universitario del Caribe. Demographic and clinical variables were recorded using a specially designed collection form, utilizing the Google Forms platform.

The study considered all patients who underwent upper limb surgery between July and September 2023 and received regional anesthesia via brachial plexus block. Eligible participants were adult of either sex, aged 18 to 80 years, with an American Society of Anesthesiologists (ASA) physical status classification of I or II [15]. Both urgent and elective surgeries of the upper limb were accepted, and the anesthetic technique required the use of a brachial plexus block. 

Exclusion criteria included patients younger than 18 years or older than 80 years, those with known allergies or hypersensitivity to local anesthetics, and patients scheduled from the outset to receive general anesthesia. Patients with preexisting neurological injury of the brachial plexus or peripheral neuropathies, local infection at the puncture site, or uncorrected coagulopathy were also excluded. Likewise, patients with severe occlusive arterial disease that could alter the studied parameters or outcomes, as well as those unable to cooperate with the procedure, were not considered for inclusion.

The purpose of the study was explained to each patient beforehand, and informed consent was obtained. Non-probability sampling was used to consecutively include the entire population that met the aforementioned criteria.

The perfusion index was recorded using a pulse oximeter connected to a Mindray iMEC-12 multiparameter monitor (Mindray, Shenzhen, China). Measurement was taken from the second finger of the hand on which the block was performed, using the basal perfusion index as a reference. Perfusion index values were collected before anesthetic blockade, as well as at five, 10, and 15 minutes after its administration. The quality of sensory block was evaluated by dermatome distribution using a two-point scale (0: normal, 1: loss of sensation to touch), compared with the same stimulation on the contralateral limb. The degree of motor blockade was also assessed using the Modified Bromage Scale for upper limbs, also at five, 10, and 15 minutes after the blockade.

Demographic variables included age, weight, height, sex, and body mass index (BMI), as well as clinical variables such as the anesthetic technique administered, the requirement for sedation and/or general anesthesia, the Bromage score, and the perfusion index.

The primary objective of this study was to determine the correlation between successful brachial plexus anesthesia and perfusion index values. The secondary outcomes included the association between anesthetic block failure and the need for sedation and/or general anesthesia.

Data collected via Google Form were exported to JAMOVI 2.3.28 for statistical analysis. Descriptive analyses were performed using absolute and relative frequencies for qualitative variables. For quantitative variables, measures of central tendency (mean: 𝑋̅ or median: Me) and dispersion (standard deviation: SD or interquartile range: IQR) were used, based on compliance with normality criteria assessed with the Shapiro-Wilk test. To compare qualitative variables between groups according to the brachial plexus block technique, the Chi-square test or, alternatively, Fisher's exact test was applied. Association analysis was performed using odds ratios (ORs), applying the Haldane-Anscombe correction when necessary.

Adjustment for potential confounders was performed through stratified analysis, considering variables such as age, weight, height, sex, BMI, and anesthetic technique used, among others. This analysis determined if the association between the dependent variable and outcomes was altered by interactions or confounders. The Mantel-Haenszel test was used, and a p-value of less than 0.05 was considered significant.

The sample was divided into four groups based on the anesthetic approach to brachial plexus block: Group 1: Interscalene approach; Group 2: Supraclavicular approach; Group 3: Infraclavicular approach; and Group 4: Axillary approach.

It is important to note that the attending anesthesiologist chose the anesthetic technique based on clinical judgment and the presence or absence of contraindications for each approach. However, to ensure methodological consistency, an anesthetic protocol was followed with 0.5% bupivacaine and 2% lidocaine, both plain, with total local anesthetic volumes between 20 and 30 cc. Blocks were performed with an echogenic needle under real-time ultrasound guidance (using a Mindray M9 ultrasound machine with a linear transducer operating at 10 MHz, automatic gain, MSK preset, and depth adjusted according to anatomical location), with visualization of hydrodissection around the target structures to confirm proper distribution of the local anesthetic.

According to Resolution 8430 of 1993 from the Colombian Ministry of Health and Social Protection regarding scientific, technical, and administrative standards for health research, this study is classified as minimal risk because it involves a surgical and anesthetic intervention that may affect the psychological, biological, physiological, or social characteristics of the participants.

## Results

A total of 82 patients from Hospital Universitario del Caribe were included in the study. Between July and September 2023, they underwent upper limb surgery, either urgently or electively. Brachial plexus block was used as the regional anesthetic technique in all cases.

The median age was 40.5 years (interquartile range [IQR]: 27.5-57.8), and the majority of patients were male (56 of 82 patients, or 68.3%). The mean weight was 67.9 kg (SD: 11.3), the mean height was 167 cm (SD: 8.38), and the mean body mass index was 24.3 (SD: 3.62) (Table 1).

**Table 1:** Sociodemographic characteristics of patients undergoing upper limb surgery with a brachial plexus block anesthetic technique

Population characteristics		
N = 82	Regional anesthesia with brachial plexus blockade	
Median age (RIQ)	40.5 (30.3)	
Median weight (SD)	67.9 (11.3)	
Median height (SD)	167 (8.38)	
Body mass index (SD)	24.3 (3.62)	
Sex	%	Frequency
Male	68.3	56
Female	31.7	26
Total	100	82

The most commonly used approach for brachial plexus block was the supraclavicular approach, applied in 56.1% of cases (46/82). This was followed by the axillary approach in 26.8% (22/82), the interscalene approach in 13.4% (11/82), and the infraclavicular approach, which was used in 3.7% (3/82) of patients (Table 2).

**Table 2:** Regional brachial plexus block technique used for upper limb surgery

Approach	Frequency	%
Interscalene	11	13.4
Supraclavicular	46	56.1
Infraclavicular	3	3.7
Axillary	22	26.8
Total	82	100

The mean perfusion index before the anesthetic block was 0.85 (SD 0.25). At five minutes after the procedure, it increased to 3.23 (SD 1.15). At 10 minutes, the mean value was 4.96 (SD 1.72), and at 15 minutes it reached 6.64 (SD 2.67) (Table 3).

**Table 3:** Perfusion index values

N = 82	Regional anesthesia with brachial plexus blockade
Before performing the block - Mean (SD)	0.85 (0.24)
5 minutes after performing the block – Mean (SD)	3.23 (1.15)
10 minutes after the block – Mean (SD)	4.96 (1.72)
15 minutes after performing the block – Mean (SD)	6.64 (2.67)

The Modified Bromage Scale for upper limb motor block revealed successful block in 8.5% (7/82) of patients at five minutes, 17.1% (14/82) at 10 minutes, and 73.2% (60/82) at 15 minutes post-procedure (Table 4). The rate of unsuccessful blocks (defined as the need for additional sedation or general anesthesia) was 24.4% (20/82). Statistically significant differences in success rates were observed when comparing the different anesthetic approaches. The supraclavicular block achieved the highest success rate, with 89.1% (41/46), followed by the axillary block with 77.3% (17/22). The infraclavicular approach demonstrated a 100% success rate, although in a limited sample of three patients. In contrast, the interscalene block showed a markedly lower success rate of 9.1% (1/11). These results suggest a significant correlation between the chosen anesthetic approach and the overall success of the block (Table 5).

**Table 4:** Modified Bromage Scale with successful scoring

Time	Frequency	%
5 minutes	7	8.5
10 minutes	14	17.1
15 minutes	60	73.2
Total	81	98.8

**Table 5:** Relationship between block type and successful anesthetic technique

Approach	Successful blocks N (%)
Interscalene	1 (9.1%)
Supraclavicular	41 (89.1%)
Infraclavicular	3 (100%)
Axillary	17 (77.3%)
Total	62/82 (75.6%)

When comparing perfusion index values greater than 3, measured 15 minutes after the block, with a two-point Modified Bromage Scale, statistically significant differences were observed, present in 82.9% (68/82) of cases. These results indicate a significant relationship between the perfusion index and motor success according to the Modified Bromage Scale (Table 6).

**Table 6:** Relationship between perfusion index 15 minutes after the anesthetic block and a score of 2 on the Modified Bromage Scale

Perfusion index value	Successful blocks N (%) (p-value < 0.001)
≤ 3	14 (18.9%)
> 3	68 (82.9%)
Total	82 (100%)

Similarly, a statistically significant difference was found when comparing a perfusion index value greater than 3 at 15 minutes with a successful anesthetic technique, with a success rate of 83.8% (62/74). This suggests a positive association between the perfusion index and the clinical outcome of the block (Table 7).

**Table 7:** Relationship between perfusion index and successful technique

Perfusion index value	Successful blocks N (%) (p-value < 0.001)
≤ 3	20 (24.4%)
> 3	62 (75.6%)
Total	82 (100%)

The comparison between the success of the Bromage scale and a successful anesthetic technique also revealed statistically significant differences: 95% (57/60) of patients with Bromage 2 achieved a successful technique, demonstrating a strong correlation between both variables (Table 8). Although the Modified Bromage Score reflects only the extent of motor block, it was selected as an objective and reproducible method for assessing block progression, as the assessment of sensory block is subjective and involves patient perception.

**Table 8:** Relationship between the Modified Bromage Scale and successful technique

Bromage Score	Successful blocks N (%)	p-value
2	57 (95%)	<0.001

Performance analysis of the perfusion index as a predictor demonstrated a sensitivity of 100%, a specificity of 40%, a positive predictive value (PPV) of 83%, and a negative predictive value (NPV) of 100%. These results suggest that a perfusion index greater than 3 after 15 minutes is highly effective in predicting successful blockade, although its moderate specificity implies that up to 60% of the cases may represent false positives. In contrast, a value lower than 3 consistently excluded anesthetic success, as reflected by the 100% NPV (Table 9). Similarly, the Modified Bromage Score analysis showed a sensitivity of 91% and a specificity of 85%, with a PPV of 95% and an NPV of 77%. At 15 minutes, a Bromage score of 2 proved to be a reliable predictor of anesthetic success, demonstrating high sensitivity and specificity while maintaining relatively low rates of false negatives (9%) and false positives (15%) (Table 10).

**Table 9:** Sensitivity and specificity of perfusion index in successful anesthetic technique

Perfusion index > 3	Successful technique N (%)		
	Yes	No	Total
Yes	62 (75.6%)	12 (14.6%)	74 (90.2%)
No	0 (0%)	8 (9.8%)	8 (9.8%)
Total	62 (75.6%)	20 (24.4%)	82 (100%)

**Table 10:** Sensitivity and specificity of the Modified Bromage Scale for successful anesthetic technique

2-point Bromage Scale	Successful technique N (%)		
	Yes	No	Total
Yes	57 (69.5%)	3 (3.7%)	60 (73.2%)
No	5 (6.1%)	17 (20.7%)	22 (26.8%)
Total	62 (75.6%)	20 (24.4%)	82 (100%)

Analysis of confounding factors

Gender was identified as a confounding factor. When the relationship between a perfusion index greater than 3 at 15 minutes and successful anesthetic technique was stratified by gender, the Mantel-Haenszel test revealed statistical significance, indicating similar odds ratios between groups and a significant association between the two variables. Therefore, gender should be considered a confounding factor in the relationship between perfusion index and anesthetic success (Table 11). In contrast, BMI did not act as a confounding factor. The Mantel-Haenszel test was not significant in this case, indicating that the odds ratios differed between groups and that no consistent association was found between BMI and the outcome of the block.

**Table 11:** Confounding factor — Mantel-Haenszel test. Sex as an influential confounding factor in the measurement of the perfusion index.

Sex	χ²	gl	p
Female	5.65	1	0.017
Male	21.83	1	< 0.001
Total	27.15	1	< 0.001

## Discussion

Determining the success of brachial plexus blockade has traditionally been based on the presence of clinical signs in the limb, assessed using the Modified Bromage Scale [10,16]. However, these signs can be ambiguous, take a long time to appear, and lack objectivity in predicting the success or failure of the block in some patients [17]. The perfusion index has been proposed as an objective method because it is an indicator of peripheral blood flow, which changes after the administration of local anesthetics [13,18,19]. When the sympathetic nervous system is stimulated, there is a decrease in the height of the pulsatile portion of the perfusion index curve, thus decreasing its value. Conversely, when the sympathetic nervous system is inhibited, the pulsatile portion of the curve increases, thus increasing the perfusion index value [14].

This study found that a perfusion index value less than 3 has an NPV of 100% in predicting failure of anesthetic brachial plexus block. Our findings and previous studies show that there is individual variation in baseline pre-block perfusion index values (range: 0.1-2.69), so the effectiveness of the perfusion index can be assessed by relative increases with respect to the baseline value [10,12-14]. Similarly, an NPV of 77% was found to predict block failure when the Modified Bromage Scale had a score of 0 or 1. However, this result may have been influenced by the latency time of the local anesthetic and the recording interval of the scale since evaluation was not performed beyond 15 minutes.

The literature reports successful ultrasound-guided brachial plexus blocks using various approaches. For instance, the interscalene approach demonstrated progressive increases in the perfusion index up to 15 minutes, rising from a baseline value of 0.2 to 2.2. With the infraclavicular approach, the baseline value was 1.8, increasing to 3.7 after 20 minutes [13,20]. Another study of 77 patients undergoing supraclavicular block found that the perfusion index increased from 2.8 to 7.1 within 30 minutes [11]. In our study, the median baseline perfusion index was 0.8, with progressive increases at five, 10, and 15 minutes, reaching a mean value of 6.64.

An increase in the perfusion index of 1.55 times the baseline value has been shown to indicate a successful axillary block after 10 minutes [12]. Similarly, a perfusion index value of 3.3 at 10 minutes after supraclavicular block, or an increase of 1.39 times the baseline value, has also been associated with success [11]. In our study, the mean perfusion index at 10 minutes was 4.96, with a 6.2-fold increase over baseline and a Modified Bromage score of 2, reflecting success in 17.1% of patients; this percentage increased to 73.2% at 15 minutes. The perfusion index was obtained through four different approaches: interscalene (13.4%), supraclavicular (56.1%), infraclavicular (3.7%), and axillary (26.8%). The supraclavicular approach was the most frequent.

In patients with failed block, minimal or no changes in the perfusion index were observed, allowing for early identification of failure and facilitating the decision to apply an alternative technique such as a repeat block or general anesthesia [12]. In our study, an unsuccessful technique associated with a perfusion index of less than 3 had a specificity of 40%. Compared with a Modified Bromage Scale of 0-1, however, the specificity was 85%. Therefore, these findings help avoid delays in the operating room, reduce costs, and improve patient satisfaction and outcomes.

The perfusion index can be consolidated as a useful tool for research in regional anesthesia, as it is an objective parameter that changes with the administration of local anesthetics and is directly related to nerve block, rather than to serum anesthetic levels [12,21]. 

One of the limitations of this study is its single-center nature, conducted in a university hospital with trained personnel. This could have influenced the block technique and its success, depending on the operator. Additionally, the relatively small sample size and short observation period may limit the generalizability of the results. However, we found a consistent relationship between increased perfusion index and brachial plexus block success across four different approaches after five minutes, making our findings worthy of reporting.

Another identified limitation was the presence of confounding bias related to sex, which affected the findings on the relationship between the perfusion index and the success of anesthetic techniques. Future studies could correct this spurious association using strategies such as restriction, randomization, and matching, as well as advanced statistical analyses, including stratification, standardization, sensitivity analysis, inverse probability weighting, and multivariate regression.

## Conclusions

The perfusion index is an objective and reliable parameter for predicting the success of brachial plexus block, showing significant increases from the first five minutes after local anesthetic administration.

In this descriptive, observational, cross-sectional study, statistical significance was established for successful brachial plexus block with a pulsatility index value greater than 3 at 15 minutes after local anesthetic administration and with a score of 2 on the Modified Bromage Scale.

A perfusion index value less than 3 was associated with a 100% negative predictive value for block failure, allowing early identification of patients with ineffective techniques and consideration of alternative strategies such as repeating the block or using general anesthesia.

The results suggest that continuous perfusion index monitoring could be integrated as a complementary tool to ultrasound and clinical assessment, optimizing real-time anesthetic decision-making.
